# Tourniquets on Railroads

**Published:** 1852-12

**Authors:** 


*Tourniquets on Railroads.*—Several of the English railroad companies, and especially the Midland, have a supply of these very important instruments on board, which are often eminently serviceable in case of accident, till a surgeon can be called. They should be kept by the conductors of our American roads also. Many a death occurs from hæmorrhage in cases of crushed limbs, wounds of blood-vessels, &c., before surgical assistance can be had. Should the public papers assist in promulgating this sentiment, the boon would soon be secured.—*Boston Med. and Surg. Journal.*

